# Abnormal sense of agency in eating disorders

**DOI:** 10.1038/s41598-023-41345-5

**Published:** 2023-08-30

**Authors:** Livia Colle, Dize Hilviu, Monica Boggio, Alessandra Toso, Paola Longo, Giovanni Abbate-Daga, Francesca Garbarini, Carlotta Fossataro

**Affiliations:** 1 Department of Psychology, University of Turin, Turin, Italy; 2 Department of Neuroscience, University of Turin, AOU Città della Salute e della Scienza, Turin, Italy; 3 MANIBUS Lab, Department of Psychology, University of Turin, Turin, Italy

**Keywords:** Psychiatric disorders, Cognitive control, Cognitive neuroscience, Motor control

## Abstract

The feeling of controlling one’s own actions and, through them, impacting the external environment (i.e. Sense of Agency—SoA) can be relevant in the eating disorders (EDs) symptomatology. Yet, it has been poorly investigated. This study aims to implicitly assess SoA exploiting the Sensory Attenuation paradigm in two groups of EDs patients (Anorexia Nervosa Restrictive and Anorexia Nervosa Binge-Purging or Bulimia Nervosa) compared to a control group. We find that controls perceive self-generated stimuli as less intense than other-generated ones showing the classic pattern of sensory attenuation. By contrast, EDs patients show the opposite pattern, with self-generated perceived as more intense than other-generated stimuli. This result indicates an alteration of the implicit component of the feeling of control in EDs patients, thus suggesting a potential implication of these results for the clinical practice and the treatment of EDs symptomatology.

## Introduction

In everyday life, we usually know what we are doing and what are the possible sensory consequences on the environment. This feeling of controlling one’s own voluntary motor acts and, through them, the course of external events is known as the *Sense of Agency*^1, 2^. According to the motor control literature, the core of the sense of agency is the link between a voluntary action and an outcome. Indeed, according to a seminal model of motor control, once motor programs are selected and sent to the periphery, an efference copy is formed and, based on this signal, a forward model predicts the sensory consequences of the movement^3^. Thus, when the actual sensory feedback corresponds exactly to the prediction, participants experience a cause-effect relationship between one’s own action and the sensory event, leading them to experience themselves as the “source of the action”^1^. Crucially, besides the subjective experience of being in charge over one’s own voluntary movements (“I voluntarily moved that body”), we are aware that those movements are being executed through our own body (“I am moving my body”)^4^. Thus, the sense of agency also implies the sense of body ownership.

The sense of agency has been found altered in different pathological conditions characterized by bodily-self awareness and/or self-control related issues, such as borderline personality disorder^5, 6^, schizophrenia^7–11^, obesity^12^ and obsessive compulsive disorder^13^. An important alteration of the bodily-self representation characterizes the symptomatology of Eating Disorders (EDs), whose development and maintenance have also been linked to the dimension of self-control^14, 15^. However, despite its clinical relevance, the dimension of self-control has remained poorly investigated, and evidence from clinical studies is limited and inconsistent^14^. Hence, in the present study, we tackled this issue by exploiting the sensory attenuation paradigm as an implicit measure of the sense of agency.

Eating disorders (EDs) are mental disorders characterized by altered eating behaviours, such as abnormal consumption or a severe and persistent restriction of food intake, that can significantly compromise the individuals’ physical and mental health and their social and professional activity^16^. Two severe conditions are Anorexia Nervosa (AN) and Bulimia Nervosa (BN). AN is characterized by a persistent restriction of calories intake with consequent weight loss, and it may occur in two different forms, namely Restricting Type or Binge-Purging Type. BN refers to recurrent episodes of binge eating followed by inappropriate compensatory behaviours^17^. The main clinical characteristics associated with EDs are negative body image, lack of self-confidence, impulsivity, perfectionism, fear of failure (performance anxiety), and self-criticism^18,19^. Besides the above-mentioned clinical features, self-control issues have been proposed to be at the root of EDs aetiology and maintenance^14,15^. In this vein, the EDs symptomatology can be understood as an attempt to compensate for an underlying sense of ineffectiveness and lack of control experienced in the rest of their life (e.g. working, relationship, parenting) that leads EDs individuals to feel intense adverse mood states, such as anger, anxiety or depression. In this regard, disordered eating and an unhealthy diet constitute a compensatory strategy to regain control, avoid negative affect associated with general life dissatisfaction and interpersonal problems, and face the negative emotional states they cannot appropriately cope with. Control over eating, body shape and weight become the primary aim, being perceived as the only successful behaviour within a context of general failures in all the other areas of functioning. Hence, their unhealthy eating behaviour is the means for achieving a feeling of being in control and, in turn, experiencing a strong sense of self^20,21^. Furthermore, EDs have a dysfunctional system for evaluating self-worth that contributes to maintaining the disorder. Indeed, whereas most people evaluate themselves based on their perceived performance in a variety of domains of life (e.g., the quality of their relationships, work, parenting, sporting ability, etc.), people with EDs judge themselves primarily, or even exclusively, in terms of their eating habits, body shape or weight and their ability to control them. Hence, their lives become centred on controlling eating, body-shape and weight to achieve thinness and avoid weight gain. These distinguishing behavioural and attitudinal characteristics are commonly shared across EDs. Indeed, longitudinal studies reported frequent cross-over among the EDs diagnostic categories, suggesting that EDs categories have much in common. They share essentially the same core psychopathology with both groups of patients over-evaluating eating, shape and weight and their control, and this psychopathology is expressed in similar attitudes and behaviors. However, while AN patients have been generally described as more harm-avoidant, neurotic, perfectionistic, and obsessive relative to controls, BN patients share many of these characteristics but tend to be more impulsive and disinhibited than those with AN^22^. How the sense of agency and the disturbed body representation are related to these different characteristics of patients with AN or BN is still a matter of investigation. A research aim for the treatment of EDs is to fully understand all the dysfunctional aspects related to the core psychopathology involved in maintaining such pathological conditions.

In recent years a growing number of studies have taken an interest in understanding the disturbed body representation in EDs, exploring whether the processing of interoceptive and exteroceptive information contributing to the generation of a coherent sense of body ownership may be altered. Both lines of research on interoceptive and exteroceptive perception in EDs are still controversial. Interoception refers to the afferent information arising from inside the body (including pain, itch, tickle, sensual touch, muscular and visceral sensations, vasomotor flush, hunger, thirst etc.) and represents the sense of the physiological state of the entire body^23^. To date, the association between interoceptive accuracy and body image distortion in people with EDs is unclear, from both clinical and experimental point of view^24–31^. For example, Pollatos and co-workers reported a lower interoceptive accuracy in AN patients compared to controls at a heartbeat detection task^32^, demonstrating also a link between reduced interoceptive accuracy and the patients’ body disatifaction^33^. However, other studies did not replicate the same results^25,34^. Exteroception is the afferent information arising from outside the body, such as visual, tactile, or auditory inputs normally occurring in the space immediately surrounding the body^35,36^. Even in the case of exteroceptive signals, data on individuals with EDs are very controversial^37–40^. For example, the Rubber Hand Illusion (RHI)^41^, has been applied to examine the bodily self in individuals acutely affected by EDs. In the study by Eshkevari and colleagues, individuals with EDs experienced the RHI significantly more strongly than controls, suggesting that the bodily self is more plastic in people with EDs^37^. Conversely, Carey & Preston^42^, exploiting a different paradigm of RHI which involved movements, reported no significant differences between EDs and controls. Therefore, the authors argued that this finding might be caused by the inclusion of voluntary movements (i.e., sense of agency).

**Figure 1:**
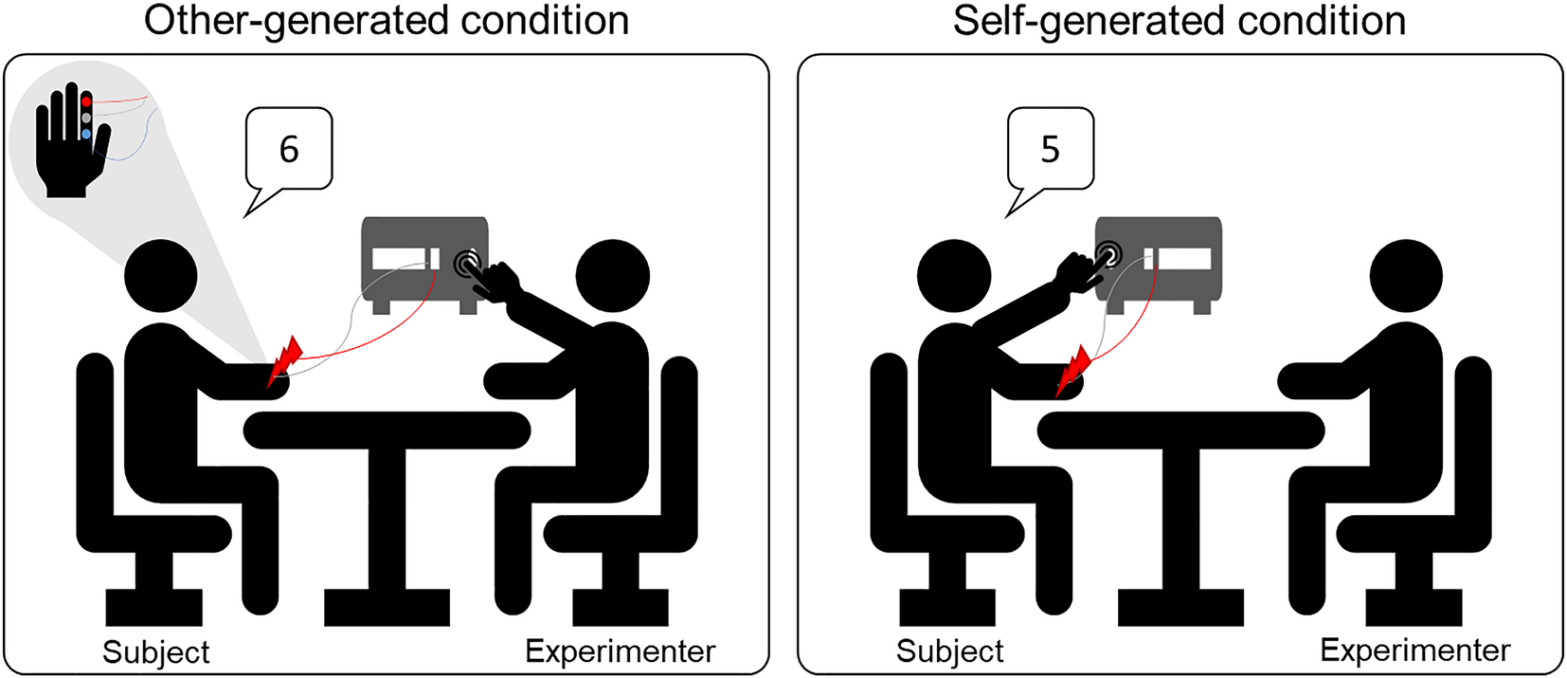
Experimental setting representation.

While greater attention has been paid to the information from inside and outside the body that jointly contributes to constructing the sense of body ownership in EDs, less attention has been given to the sense of agency. Only one study sought to implicitly measure sense of action’s control in AN^43^. In this study, the sense of agency was measured through the intentional binding task, which measures the perceived time of an action and its sensory outcome. When participants are under the impression that they cause the tone, a temporal binding occurs, and participants estimate closer the time of action and the occurrence of the tone. However, no differences in the temporal binding task between individuals with AN and controls have been found. Being the sense of agency a complex construct, it could be that using different paradigms to implicitly measure the sense of agency might allow exploring and highlighting other aspects of the feeling of control in EDs. The present study aims to exploit another well-known paradigm to implicitly assess the sense of agency: the sensory attenuation^1,10,44–46^. Such a paradigm consists in comparing the participants’ intensity perception of tactile stimuli delivered by themselves (self-generated condition) with that one of stimuli delivered by other individuals (other-generated condition), see Fig. 1. This task gives rise to the so-called sensory attenuation phenomenon in which the intensity of self-generated stimuli is perceived as significantly attenuated compared to the same stimuli generated by someone’ else. This phenomenon occurs because people automatically anticipate the sensory consequences of self-generated actions, explaining why people are unable to tickle themselves^45,47–51^. Within the motor control literature, there is a wide agreement that sensorimotor predictions affect the perception of sensory stimuli. Indeed, it is a common experience that, when we voluntarily move a part of our body, we can easily predict the sensory consequences of one’s own actions. Thus, a perfect match between predicted and actual sensory consequences of one’s own actions attenuates the perceived intensity of self-generated stimuli with respect to other-generated stimuli.

Against this background, the present study exploited the sensory attenuation paradigm to explore whether and to what extent the sense of agency in individuals with EDs is altered. To this aim, two groups of patients with EDs (Anorexia Nervosa Restrictive Type, AN-R and Anorexia Nervosa Binge-Purging Type or Bulimia Nervosa, AN-BP/BN) were compared with a group of controls.

It might be plausible that symptoms or stable traits typical of different disorders affect the sense of agency.

Our first hypothesis is that individuals with EDs would report a different trend in the sensory attenuation paradigm compared to controls. Due to the distorted bodily self-awareness and the common dysfunctional system of self-worth based on the control of eating, body shape and weight, we expect that EDs patients will have more difficulties in predicting the sensory consequence of one’s own actions, thus leading to an altered pattern of intensity perception of self-generated stimuli relative to controls. More specifically, we may anticipate the absence of the classic pattern of sensory attenuation in the EDs either because they do not judge the intensity of self- and other-generated stimuli differently or because they judge the self-generated stimuli as more intense than other-generated ones.

As second hypothesis, we expect that such confusion in the sense of agency can be strictly correlated to various aspects of symptomatic and stable traits which characterize EDs. We know, for example, that subjective body experience may be tightly related to mood disorders or other personality dysfunctions, such as impulsivity^52–54^. An individual who feels unable to initiate any kind of action, suffering from depressive symptoms, is very different from an individual who suffers from a high level of impulsivity. Therefore, subjective experience of the sense of agency combined with temperamental and symptomatic aspects may be both important contributors to the experiential aspects of the body image disturbance in EDs^55^. In terms of states that can be related to EDs, we measured in the present study depression, anxiety, dissociation, non-suicidal self-injurious behaviours, while in terms of traits, we analysed impulsivity, emotion dysregulation, identity representation, and history of traumatic experiences. Finally, we have also explored whether the body mass index (BMI) could have a significant role in driving the sense of agency in participants with EDs.

Therefore, based on the different traits that characterize AN-R and AN-BP/BN, we can anticipate a difference in the sensory attenuation pattern between the two groups, with AN-BP/BN patients more likely to fail in predicting the sensory consequences of their own actions than AN-R, because of their marked impulsive and disinhibited traits.

## Results

### Sensory attenuation results

**Figure 2:**
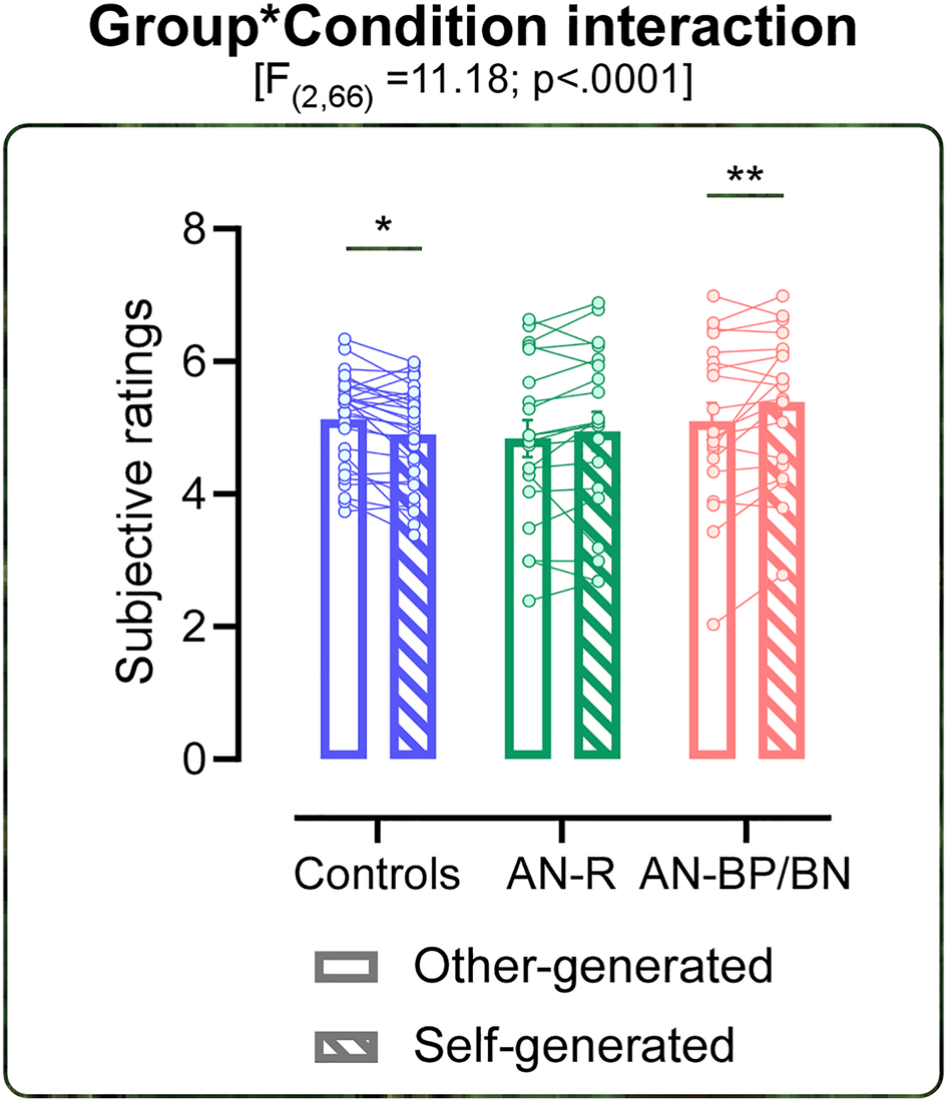
Sensory attenuation results. The figure represents the significant Group by Condition interaction. For each group (Controls in blue, AN-R in green, and AN-BP/BN in pink) the empty histograms represent the other-generated condition while the dotted ones represent the self-generated condition. Error bars indicate sem. Asterisk indicates the significant comparison (*p < 0.05; **p < 0.005). Dots represent individual values.

The ANOVA over the stimulation intensity does not show any significant differences among groups (F_(2,66)_ = 0.712; *p* = 0.494). Thus, being the somatosensory perception comparable among groups one can rule out that any difference in intensity perception was due to alteration in the elaboration of the somatosensory stimuli. The ANOVA on subjective ratings shows a significant Group by Condition interaction (F_(2,66)_ = 11.180; *p* = 0.00007; η^2^_p_ = 0.253) suggesting different patterns among the three groups (i.e. HC, AN-R and AN-BP/BN). Indeed, the HC group rates the stimulation intensity in the self-generated as significantly attenuated compared to the other-generated condition (mean ± sd: self-generated = 4.90 ± 0.75; other-generated = 5.13 ± 0.70; *p* = 0.036). While AN-BP/BN group perceives self-generated stimulations as significantly more intense than the other-generated ones (self-generated = 5.39 ± 1.09; other-generated = 5.10 ± 1.24; *p* = 0.002). The AN-R group do not report self-generated stimulations as significantly different compared to the other-generated ones (self-generated = 4.95 ± 1.32; other-generated = 4.84 ± 1.24; *p* = 0.39), (Fig. 2). Neither the main effect of group (F_(2,66)_ = 0.61; *p* = 0.54) nor the main effect of condition (F_(1,66)_ = 1.44; *p* = 0.23) are significant.

### Self-report questionnaires results

Results of self-report questionnaires are resumed in Table 1. The only questionnaire that has not a significant group effect is the CTQ but it is worth noticing that this test was completed by a reduced number of participants.

**Table 1:** Means, standard deviations and comparisons for all self-report questionnaires.

Self-report Questionnaire	AN-R (*N*), *M* ± *SD*	AN-BN/BP (*N*), *M* ± *SD*	HC (*N*), *M* ± *SD*	McDonald/Cronbach coefficients	Statistics*	Post-hoc comparisons
IDEA	(17), 2.12 ± 0.70	(18), 2.34 ± 0.98	(29), 0.648 ± 0.396	ω = 0.96	F_(2,27.165)_ = 49.603^a^, p < 0.0001	AN-R vs HC (*p* < 0.0001) AN-BN/BP vs HC (*p* < 0.0001) AN-R vs AN-BN/BP (*p* = 0.725)
EDI 2	(20), 102.6 ± 48.19	(20), 126.7 ± 50.03	(29), 39.903 ± 45.949	ω = 0.95	χ^2^_(2)_ = 31.545^b^, p < 0.0001	AN-R vs HC (*p* < 0.0001) AN-BN/BP vs HC (*p* < 0.0001) AN-R vs AN-BN/BP (*p* = 0.742)
BDI-II	(20), 17.58 ± 8.87	(20), 22.28 ± 8.03	(29), 8.065 ± 8.555	ω = 0.97	χ^2^_(2)_ = 26.379^b^, p < 0.0001	AN-R vs HC (p = 0.002) AN-BN/BP vs HC (*p* < 0.0001) AN-R vs AN-BN/BP (p = 0.531)
ISAS						
Interpersonal	(20), 5.50 ± 7.11	(20), 12.05 ± 9.75	(29), 0.581 ± 1.876	ω = 0.90	χ^2^_(2)_ = 31.369^b^, p < 0.0001	AN-R vs HC (*p* = 0.010) AN-BN/BP vs HC (*p* = 0.000) AN-R vs AN-BN/BP (*p* = 0.053)
Intrapersonal	(20), 8.20 ± 9.2	(20), 16.85 ± 9.28	(29), 1.32 ± 3.83	ω = 0.95	χ^2^_(2)_ = 30.177^b^, p < 0.0001	AN-R vs HC (*p* = 0.031) AN-BN/BP vs HC (*p* < 0.0001) AN-R vs AN-BN/BP (*p* = 0.025)
STAI-Y						
State	(20), 57.65 ± 12.71	(20), 63.95 ± 8.88	(29), 36.71 ± 12.10	α = 0.95	F_(2,68)_ = 39.968^c^, p < 0.0001	AN-R vs HC (*p* < 0.0001) AN-BN/BP vs HC (*p* < 0.0001) AN-R vs AN-BN/BP (*p* = 0.069)
Trait	(20), 61.65 ± 9.62	(20), 68.15 ± 7.79	(29), 42.61 ± 11.44	α = 0.89	F_(2,43.416)_ = 45.591^a^, p < 0.0001	AN-R vs HC (*p* < 0.0001) AN-BN/BP vs HC (*p* < 0.0001) AN-R vs AN-BN/BP (*p* = 0.062)
DES	(19), 22.32 ± 14.40	(18), 35.61 ± 20.32	(29), 12.14 ± 7.37	ω = 0.93	F_(2,28.046)_ = 13.715^a^, p < 0.0001	AN-R vs HC (*p* = 0.023) AN-BN/BP vs HC (*p* < 0.0001) AN-R vs AN-BN/BP (*p* = 0.073)
BIS-11	(20), 59.65 ± 7.21	(20), 65.05 ± 23.78	(29), 60.19 ± 10.87	ω = 0.97	χ^2^_(2)_ = 6.542^b^, p < 0.0001	AN-R vs HC (*p* = 1.000) AN-BN/BP vs HC (*p* = 0.047) AN-R vs AN-BN/BP (*p* = 0.125)
DERS	(19), 111.42 ± 19.66	(18), 124.11 ± 22.7	(29), 82.13 ± 25.37	ω = 0.89	F_(2,65)_ = 21.070^c^, p < 0.0001	AN-R vs HC (*p* = 0.000) AN-BN/BP vs HC (*p* = 0.000) AN-R vs AN-BN/BP (*p* = 0.079)
CTQ	(12), 26.67 ± 2.19	(11), 26.46 ± 3.56	(23), 25.48 ± 3.20	ω = 0.84	F_(2,43)_ = 0.735^c^, p = 0.489	

*IDEA* identity and eating disorders; *EDI*-*2* eating disorder inventory, version 2; *BDI-II* beck depression inventory II; *ISAS* inventory of statements about self-injury; *STAI*-*Y* state-trait anxiety inventory; *DES* dissociative experiences scale; *BIS*-*11* Barratt impulsiveness scale; *DERS* difficulties in emotion regulation scale; *CTQ* childhood trauma questionnaire.

*Different statistics were used following the assumptions for the distribution of each self-report test,

^a^Kruskal-Wallis test and Dunn’s post-hoc test,

^b^One-way ANOVA and Bonferroni post-hoc test,

^c^One-way ANOVA and Newman-Keuls post-hoc test.

### Correlation results

Spearman correlations between BMI and self-report questionnaire results considering only participants with EDs and attenuation indexes are presented in Table 2. No significant correlations emerged.

**Table 2:** Spearman correlation results between the self-report questionnaires and the attenuation indexes in all participants (Anorexia Nervosa Restrictive, Anorexia Nervosa Binge/Purging and Bulimia Nervosa).

Self-report questionnaire	*N*	*r*_*s*_	B-H *p*_*adj*_ values
IDEA	35	0.090	0.607
EDI 2	40	− 0.083	0.609
BDI-II	40	− 0.165	0.309
ISAS			
Interpersonal	40	− 0.072	0.657
Intrapersonal	40	− 0.027	0.868
STAI-Y			
State	40	− 0.167	0.302
Trait	40	− 0.186	0.250
DES	37	0.244	0.145
BIS-11	40	0.177	0.274
DERS	37	− 0.153	0.366
BMI	40	− 0.269	0.093

*IDEA* identity and eating disorders, *EDI*-*2* eating disorder inventory, version 2, *BDI*-*II* beck depression inventory II, ISAS inventory of statements about self-injury, STAI-Y state-trait anxiety inventory, DES dissociative experiences scale; *BIS*-*11* barratt impulsiveness scale, *DERS* difficulties in emotion regulation scale.

## Discussion

Characterizing disturbances in the need for control among individuals with EDs is critical to understand the pathophysiology of these conditions, as both the relentless pursuit of an unhealthy body weight and the disordered eating behaviours that typify EDs. Previous research has demonstrated that disturbed eating behaviour can be correlated, in part, by a desire to alter subjective body experience and not merely body appearance^56, 57^.

The present study investigated a specific aspect of subjective body experience, which is the sense of agency, within EDs individuals and healthy controls. Specifically, for the first time the sensory attenuation paradigm was employed as implicit measure of the sense of agency to evaluate it in EDs individuals and address its relationship with symptoms and traits of the disorders.

Results revealed that, while HC group showed the classical sensory attenuation pattern, with self-generated stimuli perceived as significantly less intense as compared to other-generated ones, EDs individuals displayed a reverse pattern. In particular, this result was significantly evident in individuals with AN-BP/BN. Indeed, AN-BP/BN individuals perceive self-generated stimulations as significantly more intense than other-generated ones. Therefore, individuals with AN-BP/BN did not show sensory attenuation in self-generated stimulations. These results, in line with evidence suggesting a broad neural dysregulation to interoceptive stimuli in subjects remitted by AN displaying an alter insula response to either pain^58^ and pleasant touch^59^ anticipation, highlight a dysregulation of exteroceptive signals.

One previous study attempted to measure the sense of agency through the moving RHI paradigm^60^ which leads participants to incorporate movement of the rubber hand. In contrast with our results, Carey and colleagues did not observe any differences between EDs and HC controls. Both groups displayed a strong sense of ownership and agency towards the fake hand in synchronous condition. Previous research with classic static RHI paradigm collected contradictory results. Eshkevari and colleagues^37^ showed that EDs participants displayed higher sense of ownership towards the fake hand, while Keizer et al.^61^ did not observe differences between EDs and HC groups. Carey and Preston argued that the lack of differences in their experimental data may account for the motor response required in the study. The authors suggested that it is difficult to dissociate feeling of agency and feeling of ownership when voluntary movement is involved, since sensory feedback of movement is likely to further enhance the sense of ownership^60^. Only one study by Engel and colleagues^43^ has evaluated the sense of agency in individuals with EDs by exploiting the intentional binding task, which is specifically devised to implicitly assess the sense of agency. Also in this case, results did not show any group differences on the sense of agency between individuals with AN and the control group. They argued that agency is a complex construct and given that the intentional binding task measures only binding over an action and its consequences, it could be a relatively predictable task. Thus, they suggested that their results might not be generalized to all aspects of agency, which can be better detected by different tasks. In line with this interpretation, the sensory attenuation paradigm resulted an effective tool to investigate the sense of agency in EDs with the advantage to implicitly measure it without the manipulation of the sense of body ownership. Measuring sense of agency independently from the sense of body ownership disconfirmed the hypothesis of Carey and Preston^60^ that sense of agency in EDs is less dysfunctional than body ownership. Our results showed that both individuals with AN-R and individuals with AN-BP/BN do not experience sensory attenuation as the HC group. In particular individuals with AN-BP/BN showed the greatest discrepancy in sensory perception between self- and other-generated stimulations, perceiving the self-generated as considerably more intense than the other-generated ones.

In contrast with our expectation, none of the psychopathological symptoms and trait measurements of the participants with EDs correlated with the sensory attenuation index. However, interestingly, the subgroup of AN-BP/BN presented more impulsivity which can be related to the reverse performance in the sensory attenuation paradigm^62^. We can hypothesize that this impulsivity, typical of individuals with AN-BP/BN, might partially explain the reduction in the sense of agency. Indeed, results obtained from the BIS-11 scale, measuring impulsivity, showed that AN-R exhibit a similar level of impulsivity compared to HC while AN-BP/BN presented the highest score. Moreover, impulsivity is strictly connected to emotional dysregulation, which was evaluated in our study through the DERS questionnaire. In this case, AN-BN/BP showed the highest score while the AN-R group presented an intermediate score between AN-BN/BP and HC.

High level of emotional responses is also evident from data collected with the STAI-Y questionnaire, both state and trait, where AN-BN/BP manifested a higher level of anxiety compared to AN-R and both groups with EDs (AN-BN/BP and AN-R) were significantly different than HC. Research in EDs has shown that there is a high prevalence of anxiety, and increased activation of cognitive control as an attempt to compensate for the impaired ability to perceive interoceptive information^63, 64^. Indeed, many of the symptoms observed in EDs could be related to deficits in interoceptive perception, such as altered subjective responses to food, pain and heart beat awareness^65–67^.

The absence of correlations between clinical measures and attenuation index suggest that the alteration of sense of agency is a relatively stable trait as already hypothesized more generally in previous studies investigating alterations of neural signals during perception in patients recovered from EDs^67, 68^.

In addition, the sense of agency was not correlated to the BMI suggesting that the results found in the present research might be independent from the weight of the participants but more related to their clinical condition.

Overall, the present findings support previous research suggesting that individuals with EDs have a more malleable experience of the bodily self. Previous studies showed that perceptual estimation of body size can be useful to improve the subjective body experience in EDs^61^. Moreover, the novel findings of the present research show that that the need for control involves also the sense of agency. Our findings rise the possibility that body image disturbances typical of EDs can be also connected with an interoceptive/exteroceptive imbalance within a lack of sense of agency. This suggests that clinical practice may potentially be improved by expanding the targets of exposure to include sensory experiences including sense of agency.

Body image disturbance has prognostic significance, predicting illness onset, maintenance, and remission^69–71^. These findings could have important clinical implication within the treatment of body image disturbance in EDs. Further investigation of the sense of agency has the potential to guide the development of novel intervention approaches to address symptom that eludes change.

The limit of the present study pertains the sample size, which is relatively small. A greater sample size that would increase the power of the analysis is required to confirm the present data and better identify any relationship between psychopathological symptoms and the alteration in the sense of agency in EDs. Further studies need to evaluate whether and to what extent the sense of agency plays a role in shaping the clinical manifestation of individuals with EDs. Given that our sample were inpatients at the time of the research, we do not have reliable information on outpatients with less acute EDs symptoms. Finally, this study did not find any influence of psychopathological states and traits on the sense agency. However, it does show some differences in the sense of agency and in different aspects of psychopathology between individuals with AN-R and AN-BN/BP. Further studies should investigate the sense of agency considering separately this two clinically different conditions of EDs.

## Methods

### Participants

A total of 69 female participants was enrolled for the present research, 29 of them as healthy control group (HC) and 40 as experimental group (EDs). Within the EDs group, 20 participants were diagnosed with Anorexia Nervosa Restrictive Type (AN-R) and 20 with Anorexia Nervosa Binge-Purging Type or Bulimia Nervosa (AN-BP/BN n. 16/n. 4), according to the criteria of the DSM-5^17^. AN-BP/BN participants were grouped in one category since they show a similar behavioural pattern in relation to the binge purging behaviours of the disorders^72^ (see also^73^ for a similar grouping method). The three groups (AN-R, AN-BP/BN and HC) showed a significant difference when comparing their BMI (F_(2,57)_ = 42.040; *p* < 0.001). At post hoc comparisons, the BMI of HC resulted significantly different compared to that one of both AN-R (p < 0.001) and AN-BP/BN (p < 0.001), and the BMI was significantly different between the two EDs groups as well (p = 0.003). However, it is worth noticing that the BMI information is available for only 20 healthy participants. No difference was detected between the two experimental groups (AN-R and AN-BP/BN) concerning the pharmacology treatment tested by the Fisher exact test (χ^2^(1) = 1.29; p = 0.22). The HC group was recruited by means of advertise on social media of the research group and by contacting a pool of volunteers which expressed their interest to be contacted for participating to experiments. The HC group had to report no history of current or previous psychiatric illness. All patients were recruited at the Eating Disorders Centre—University of Turin, AOU Città della Salute e della Scienza in Turin, Italy. Substance and/or alcohol abuse or dependence were considered as exclusion criteria. Furthermore, patients had not to be affected by schizophrenia, schizo-affective disorder, bipolar disorder or organic mental syndrome. No significant distinction was observed in the age among the three groups (HC, AN-R and AN-BP/BN; F_(2,66)_ = 3.061; *p* = 0.054). However, they differentiate at the educational level (F_(2,66)_ = 28.488; *p* < 0.001) since the HC group present a higher number of years of education compared to the other two groups. All subjects signed the informed consent. In accordance with the Declaration of Helsinki, the experimental procedure was approved by local ethics committee of both the University of Turin (Prot. n. 3167, 1/02/2016) and Città della Salute e della Scienza (Prot. n. 0017116, 13/02/2019). See Table 3 for demographical and clinical information.

**Table 3:** Participants’ demographic information.

Group label	*N*	Age	Educational level	Body mass index	Pharmacological treatment	
		*M* ± *SD*	*M* ± *SD*	*M* ± *SD*	Frequency	(Type of drugs, Frequency)
HC	29	26.21 ± 4.69	17.17 ± 1.89	20.45 ± 2.42*	–	
AN-R	20	22.55 ± 7.32	13.15 ± 1.98	14.31 ± 1.96	14	AD = 12, BDZ = 9 AP = 4, STAB = 1
AN-BP/BN	16/4	23.10 ± 4.98	13.50 ± 2.46	16.40 ± 2.05	17	AD = 11, BDZ = 13 AP = 12, STAB = 3

*HC* healthy controls, *AN*-*R* anorexia nervosa restrictive, *AN*-*BP*/*BN* anorexia nervosa binge/purging and bulimia nervosa, *AD* Antidepressant, *BDZ* benzodiazepines, *AP* antipsychotics, *STAB* mood stabilizers.

*BMI of 9 participants of the HC groups is missing.

### Experimental procedure

During the experiment, participants were in a familiar room, seated on a chair in front of a desk, and they laid their hands palm down. Note that, the participant and the co-experimenter were seated in front of each other as in previous experiments adopting the very same sensory attenuation paradigm (see for e.g.,^5,12,74,75^). Tactile stimuli were delivered in two experimental conditions differing from whether the button delivering the stimulation was pressed by either the participants’ left hand (i.e., *self-generated* condition) or the experimenter (i.e., *other-generated* condition) (Fig. 1). A vocal instruction indicated if the participant (self-generated instruction) or the experimenter (other-generated instruction) had to press the button triggering the stimulation. Note that the delivered stimulation was always at the same intensity and participants did not received any specific information about the stimulation intensity employed during the experiment. After each stimulation, participants were asked to rate how much intense they perceived the stimulation using a 0–7 points Likert’s Scale where 0 corresponded to “no stimulation” and 7 corresponded to “very high intense stimulation”. A total of 44 stimulations were delivered in a random order: 20 *self-generated stimulations*, 20 *other-generated stimulations* and 4 catch trials (i.e., trials without stimulation to avoid a central tendency effect or response biases and to monitor possible phantom sensations) that afterwards were excluded from the data analysis.

### Electrical stimulation

Tactile stimulations were transcutaneous electrical stimuli consisting in constant current square-wave pulses (Digitimer, Model DS7A) delivered using a couple of surface bipolar electrodes attached on the index finger of the participant’s right-hand. In order to avoid habituation two slightly different sites were stimulated (see Fig. 1). To this aim, three electrodes were attached on the participants’ finger, one electrode with negative polarity and two electrodes with positive polarity (one in a more proximal position, the other one in a more distal position, on the phalanx). The two couples of electrodes were alternated randomly by changing their configuration on the stimulator so that participants were unaware of this changes. This procedure gave to participants the illusion that intensity could randomly change as well as the stimulation could be felt on distinct part of the finger. Stimulation intensity was set according to each participant’s sensory threshold level (i.e., when participants were able to detect stimuli in the 50% of trials) and its duration was 200 μs. Mean intensity thresholds were 1.49 ± 0.40 mA, range 0.55–2.64 mA for the HC, 1.40 ± 0.39 mA, range 0.91- 2.18 for AN-R group, and 1.35 ± 0.44 mA, range 0.23–2.08 mA for the AN-BP/BN group. To ensure that participants could always perceive the stimulation, during the experimental session the intensity was increased using the formula: *stimulation intensity* = intensity threshold*2.5 mA.

### Self-report questionnaires

Different measures on several psychological dimensions were collected through the completion of a set of self-report questionnaires. Some of these questionnaires were specifically focused on eating behaviours, i.e., IDEA and EDI 2. Other psychiatric symptoms were assessed through further questionnaires. A brief and introductive description for each test is provided:

- *Identity and eating disorders*—IDEA^76^ for the clinical assessment of abnormalities about identity’s perception and experience of the perception of one’s own body. The questionnaire explores how different variables such as the gaze of others, the dietary restriction and objective features (e.g., weight) can alter and impact the lived and inner perception of participants’ body. Moreover the tool investigates the sense of alienation from the body, commonly reported by patients with eating disorders.
- *Eating disorder inventory 2*—EDI 2^77^ to assess psychological features associated with eating disorders. It comprises eleven sub-scales, which include: drive for thinness, bulimia, body dissatisfaction, perfectionism, interpersonal distrust, interoceptive awareness, maturity fears, ineffectiveness, asceticism, impulsiveness and social insecurity.
- *Beck depression inventory*—BDI^78^ measures the incidence and severity of depressive symptoms, investigating both the cognitive-affective component and the somatic component. The BDI is composed of 21 items to which the subject responds on a 4-point Likert scale (with a range from 0 to 3). Questions are based on how he/she felt in the previous two weeks about specific areas of daily life: sadness, pessimism, sense of failure, loss of pleasure, guilt, feelings of punishment, self-esteem, self-criticalness, suicidal thoughts, crying, agitation, loss of interest, indecision, sense of worthlessness, loss of energy, changes in sleeping, irritability, changes in appetite, concentration, fatigue, and loss of libido.
- *Inventory of statements about self-injury* – ISAS^79^ for the evaluation of self-harming behaviour. In the first section of this questionnaire, the subject is asked about the frequency and nature of self-injurious behaviour. The second section examine the motivations behind these behaviours are investigated through two main factors of self-harm: *interpersonal factors* (autonomy, interpersonal boundaries, interpersonal influence, peer-bonding, revenge, self-care, sensation-seeking and toughness), and *intrapersonal factors* (affect regulation, anti-dissociation, anti-suicide, marking distress and self-punishment). There are 39 items characterized by a 3-point Likert scale, where 0 = not relevant to my experience and 3 = very relevant to my experience. In the third, and last, section of the questionnaire, subjects can describe in more detail his/her own experiences regarding the functions investigated in the previous section.
- *State-trait anxiety inventory* – STAI-Y^80^ for the evaluation of anxiety. This questionnaire consists of two sub-scales: Y1, which investigates state anxiety (i.e. how the individual feels in the specific moment of the administration of the questionnaire, and describes his/her current moods), while Y2 evaluates trait anxiety (i.e. the participants’ usual mood, their stable and persistent emotional state). Both scales contain 20 items, and the score is assigned on a 4-point Likert’s scale in which 1 corresponds to “not at all” and 4 to “very much”.
- *Dissociative experiences Scal*e –DES^81^ for the evaluation of dissociative events, their severity and their typology. It is composed of 28 items that describe the most common dissociative experiences. Subjects have to rate how frequently each of these experiences has occurred over the course of his/her life by using a 11-point Likert’s scale, which proposes a percentage from 0 at 100%. According to the literature, a global score above 30 could suggest a dissociative disorder^82^.
- *Barratt impulsiveness scale 11*—BIS 11^83^ to assess emotion dysregulation and impulsive traits. It allows the identification of six first-order factors and three second-order factors: the first-order factors attention and cognitive instability identify *attentional impulsiveness*; perseverance and motor behaviour denote *cognitive impulsiveness* and self-control and cognitive complexity specify *unplanned impulsiveness*. This tool is composed of 30 items evaluated on a 4-point Likert’s scale, where scores correspond to: 1 = never/rarely and 4 = almost always/always.
- *Difficulties in emotion regulation scale*—DERS^84^ to assess emotion dysregulation by four dimensions: awareness and understanding emotions, acceptance of emotions, ability to engage in goal-directed behaviours when experiencing negative emotions, and access to emotion regulation strategies perceived as effective. This scale is composed of 36 items scored on a 5-point Likert’s scale where 1 corresponds to “almost never” (0–10%), 2 to “sometimes” (11–35%), 3 to “about half the time” (36–65%), 4 to “many times” (66– 90%) and 5 to “almost always” (91–100%).
- *Childhood trauma questionnaire* – CTQ^85^ for the assessment of the presence of any traumatic experiences in childhood. The questionnaire analyses five kinds of abuse: sexual abuse, physical abuse, psychological abuse, physical neglect and emotional neglect. There is also the MID/IU index, which indicates the possibility of denied or minimized abuses.

### Data analysis

During catch trials, all participants gave a rating of zero, thus excluding the presence of response bias and false alarms. Firstly, to test whether the somatosensory perception was comparable among the three groups (HC, AN-R and AN-BP/BN), a repeated measures ANOVA was performed on the stimulation intensity in mA. Secondly, subjective ratings were analysed by means of a repeated measures ANOVA with *Group* (three levels: HC, AN-R and AN-BP/BN) as between subject factor and *Condition* (two levels: Self-generated, Other-generated) as within subject factor. Post-hoc comparisons were performed with the Bonferroni test. We checked the normal distribution of the residuals in each sub-groups by means of Shapiro–Wilk test 0.938 > W > 0.960; 0.4 > *p* > 0.09).

To explore any significant difference among the three groups on the scores to the different questionnaires (IDEA, EDI 2, BDI-II, ISAS, STAI-Y, DES, BIS-11, DERS, and CTQ) we ran a series of one-way ANOVA with group (three levels: AN-R, AN-BP/BN and HC) as between subjects factor after checking for assumptions (otherwise Kruskal–Wallis was used) and an appropriate post-hoc test was chosen for each comparison. It is worth noting that questionnaires were not completed by all participants (see *N* in Table 2), therefore the analysis for some questionnaires were performed on reduced samples. McDonald’s omega (ω) or Cronbach alpha (α) coefficients of reliability were calculated (See Table 2).

Finally, in order to investigate possible relations between BMI, psychological aspects evaluated by the self-report questionnaires and the sensory attenuation, we performed Spearman correlations between BMI, self-report questionnaires results and the attenuation index. To this aim, we calculated an *attenuation index* (∆) by subtracting the mean ratings given at the other-generated condition from the mean ratings provided in the self-generated condition (Δ_n_ = S_n_–O_n_; S: mean of the self-generated ratings of subject *n*; O: mean of the other-generated ratings of subject *n*; Δ = attenuation index of subject *n*). If necessary, the significance level (*p* value) was corrected using a false discovery rate (FDR) procedure^86^. Correlations were performed only on participants with EDs (i.e. excluding HC group) in order to rule out that relations between symptoms and the index of attenuation could be due to an effect of the groups rather than to a close correlation with a gradient of severity of psychopathology.

## Data Availability

The datasets used and/or analysed during the current study are available in the Mendely repository, https://data.mendeley.com/datasets/2ynfcyfmtc/draft?a=6e2bb201-ae15-4191-a2d3-7de37a45d8d4.
